# Generative design of large-scale fluid flow structures via steady-state diffusion-based dehomogenization

**DOI:** 10.1038/s41598-023-41316-w

**Published:** 2023-09-01

**Authors:** Sarah N. Hankins, Yuqing Zhou, Danny J. Lohan, Ercan M. Dede

**Affiliations:** Electronics Research Department, Toyota Research Institute of North America, 1555 Woodridge Avenue, Ann Arbor, MI 48105 USA

**Keywords:** Mechanical engineering, Fluid dynamics

## Abstract

A computationally efficient dehomogenization technique was developed based on a bioinspired diffusion-based pattern generation algorithm to convert an orientation field into explicit large-scale fluid flow channel structures. Due to the transient nature of diffusion and reaction, most diffusion-based pattern generation models were solved in both time and space. In this work, we remove the temporal dependency and directly solve a steady-state equation. The steady-state Swift-Hohenberg model was selected due to its simplistic form as a single variable equation and intuitive parameter setting for pattern geometry control. Through comparison studies, we demonstrated that the steady-state model can produce statistically equivalent solutions to the transient model with potential computational speedup. This work marks an early foray into the use of steady-state pattern generation models for rapid dehomogenization in multiphysics engineering design applications. To highlight the benefits of this approach, the steady-state model was used to dehomogenize optimized orientation fields for the design of microreactor flow structures involving hundreds of microchannels in combination with a porous gas diffusion layer. A homogenization-based multi-objective optimization routine was used to produce a multi-objective Pareto set that explored the trade-offs between flow resistance and reactant distribution variability. In total, the diffusion-based dehomogenization method enabled the generation of 200 unique and distinctly different microreactor flow channel designs. The proposed dehomogenization approach permits comprehensive exploration of numerous bioinspired solutions capturing the full complexity of the optimization and Swift-Hohenberg design space.

## Introduction

Computational efficiency, structural performance, and manufacturability continue to drive the field of topology optimization. In particular, the density-based method has become popular in the field for research and industrial applications^1^. The method is well-known for its ability to generate designs that meet both the loading and fabrication requirements for an array of applications in areas such as structural mechanics^2^, fluid flow^3^, and heat transfer^4^. However, large giga-resolution design domains, such as bridges^5^ and airplane wings^6^, require thousands of CPU cores (i.e., access to supercomputers) to perform density-based topology optimization in an acceptable amount of time. Additionally, for fluid flow structure design, explicit optimization involving only ~ 50 channels becomes “large-scale” computationally, necessitating millions of elements and solution on a GPU^7^; this is due to the high resolution required to adequately resolve pressure and velocity fields across the entire design space. As a result, numerous research efforts have been aimed at developing innovative approaches that can overcome such limitations.

The well-established homogenization-based method^8^ has recently resurfaced as a computationally efficient option to design high-resolution structures when paired with novel dehomogenization techniques. For methods that assemble 2D square (3D cube) unit cells with parameterized geometries^9–11^ (such as square, rectangular, circular, and crossbar) and non-parametric cell geometries^12^, readers are referred to^13^ for a comprehensive review. The connectivity across the interface of adjacent cells can pose challenges if distinct topologies are allowed in the cell level^13^.

The homogenization-based method is generally not used as a stand-alone design tool due to homogenization solutions that cannot be easily manufactured. The post-processing step to generate explicit microscale geometries such that they match the optimized homogenized property is known as dehomogenization. For periodic unit cells, this process is also previously known as inverse homogenization^14^. Such a two-step design approach is appealing because the homogenization-based optimization can be performed on a coarse mesh, reducing the computational cost, while the dehomogenization can be performed on a fine mesh to obtain explicit designs with high-resolution intricate details.

To obtain the well-connected high-resolution design from a spatially varying homogenization design defined by a scalar field and/or an orientation field, a projection-based post-processing method was proposed^15^, followed by recent simplifications and improvements^16,17^. The projection-based dehomogenization concept has also been extended to 3D problems^18,19^ and a convolutional neural network implementation^20^. An important requirement for the projection-based dehomogenization is that the spatially varying orientations are smooth throughout the domain^13^.

In parallel, an alternative approach has emerged which uses pattern generation algorithms, derived from work by Alan Turing^21^, to develop a partial differential equation (PDE) based class of bioinspired dehomogenization solutions for engineering design applications^22–29^, just to name a few. Among them, a fluid flow experiment was conducted to validate the dehomogenized microchannel design^30^. Many pattern generation algorithms are rooted in Turing’s theory of a reaction–diffusion system that models the interaction of two chemical species (or morphogens)^21^. Mathematically, this model can be represented by a system of coupled PDEs that describe the evolution of the chemicals in both time and space. Reaction–diffusion models generally create patterns using the fundamental concept of local-activation and long-range inhibition (LALI). In the two-system model, the slowly diffusing activator morphogen promotes the production of itself along with the inhibitor morphogen. This creates regions with high concentration of activator species. The purpose of the rapidly diffusing inhibitor morphogen is to ensure that the high concentration regions are separated by a distance, defined by the diffusion rates of the two chemical species, which creates the formation of periodic patterns^31,32^.

Reaction–diffusion models provide a clear and intuitive understanding of the LALI pattern generation mechanism. However, there is a simpler model that encompasses the LALI mechanism in a single variable PDE known as the Swift-Hohenberg equation^33^. The model takes advantage of the fact that the activator chemical in the reaction–diffusion system is the primary driver of the pattern generation process. This is because the morphogen not only activates itself locally, but it also indirectly inhibits itself due to the simultaneous production of the inhibitor morphogen. Therefore, the Swift-Hohenberg equation models the cumulative effect of the local activation and long-range inhibition using a single variable PDE that evolves in time and space^31^. Within the equation, a fourth order gradient operator is used to capture the long-range features, while a second order gradient operator is used to capture the short-range features^27^. Despite its simplicity, the LALI logic embedded within the equation enables the model to produce periodic patterns like those found in more complex reaction–diffusion systems, as shown herein. Nonetheless, exhaustive studies may reveal that the equation may not be capable of modeling all conceivable pattern generation phenomena, and in some cases different models might still be required^27,31^.

The purpose of this paper is to demonstrate a computationally efficient approach for novel bioinspired diffusion-based dehomogenization of multiphysics microreactor channel structures. Pattern generation models can exploit an anisotropic diffusion tensor such that structural elements emerge according to the prescribed orientation field. Here, we work with the anisotropic permeability of a porous flow medium in contrast to the fiber angle in prior solid mechanics research^27^. As a result, the process of diffusion promotes continuous flow channels, for our microreactor problem at hand, and a seamless transition between features despite the complexity of the design domain. These characteristics are geometrically distinct and can be difficult to achieve without a diffusion-based model. Intrinsically, diffusion is conceived as a process that evolves in time and space. Therefore, it is natural to solve bioinspired diffusion-based dehomogenization in time and space, as in the work^22,23,25,27,29^. However, the temporal process may be computationally time consuming and diminish the usefulness of the tool in the early concept generation phase. To overcome this barrier, we propose removing the temporal domain by directly solving the steady-state single variable Swift-Hohenberg equation.

Through several numerical experiments, our results reveal that the steady-state Swift-Hohenberg model generally converges faster than a transient diffusion-based model without significant tuning of solver parameters. The dehomogenization time of steady-state cases range from 70 to 124 s for a fluid flow channel design problem involving hundreds of microchannels. To highlight the efficiency of the proposed technique when newly applied to our multiphysics problem, a Pareto set was developed, using a homogenization-based optimization routine, to explore the design tradeoffs between pressure drop and reactant distribution variability for microreactor flow channels including a porous gas diffusion layer. In total, 200 distinctly different microreactor flow channel designs were generated using the steady-state dehomogenization technique. The designs span the multi-objective optimization space as well as the design space that is unique to the Swift-Hohenberg model to obtain relevant flow control features such as pin fins, channels, and hybrid structures beneficial for fluid flow problems. As a further contribution, it was demonstrated that this method can be effectively employed to design functionally graded channel structures for microreactors comprising multiple zones with spatially varying physical length scales. Thus, this paper highlights an early foray in the use of a steady-state pattern generation model as a rapid generative engineering design tool for large-scale, multiphysics, fluid flow applications.

## Results

**Figure 1:**
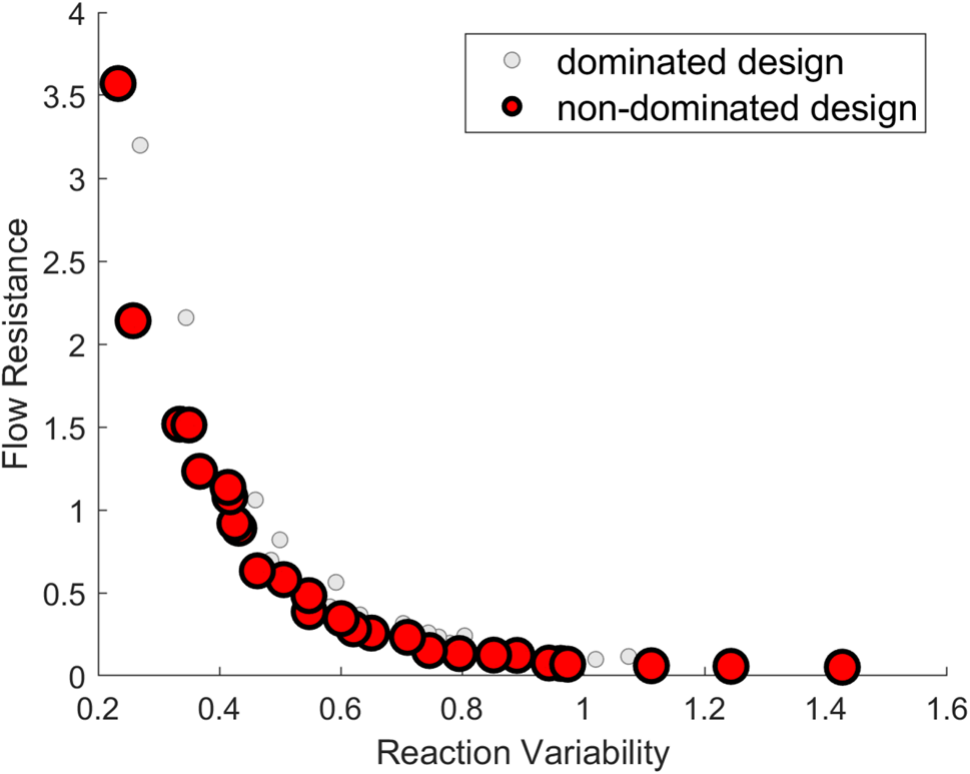
Multi-objective Pareto set generated from the homogenization-based optimization routine. Plotted are the normalized reactant distribution variability (X axis) versus the normalized flow resistance values (Y axis) for different weighting schemes defined within the objective function. Larger red circles indicate non-dominated Pareto optimal solutions. Smaller gray circles indicate dominated solutions.

A multi-objective Pareto set for the design of microreactor flow channels was developed by performing a grid search on a multi-objective weighting scheme for the optimization problem of interest. The weights were specified with a linear interval spacing of 0.02. In our problem, the first objective function term weight, ${w}_{1}$, controls the reactant distribution variability, while the second objective function term weight, ${w}_{2}$, controls the flow resistance; refer to Eq. (1 )in Methods. Figure 1 reveals a grid search results for our multi-objective optimization problem with non-dominated Pareto optimal designs labeled in red. The Pareto set illustrates how the flow resistance and reactant distribution variability change as the objective function’s weighting scheme is altered during optimization.

**Figure 2:**
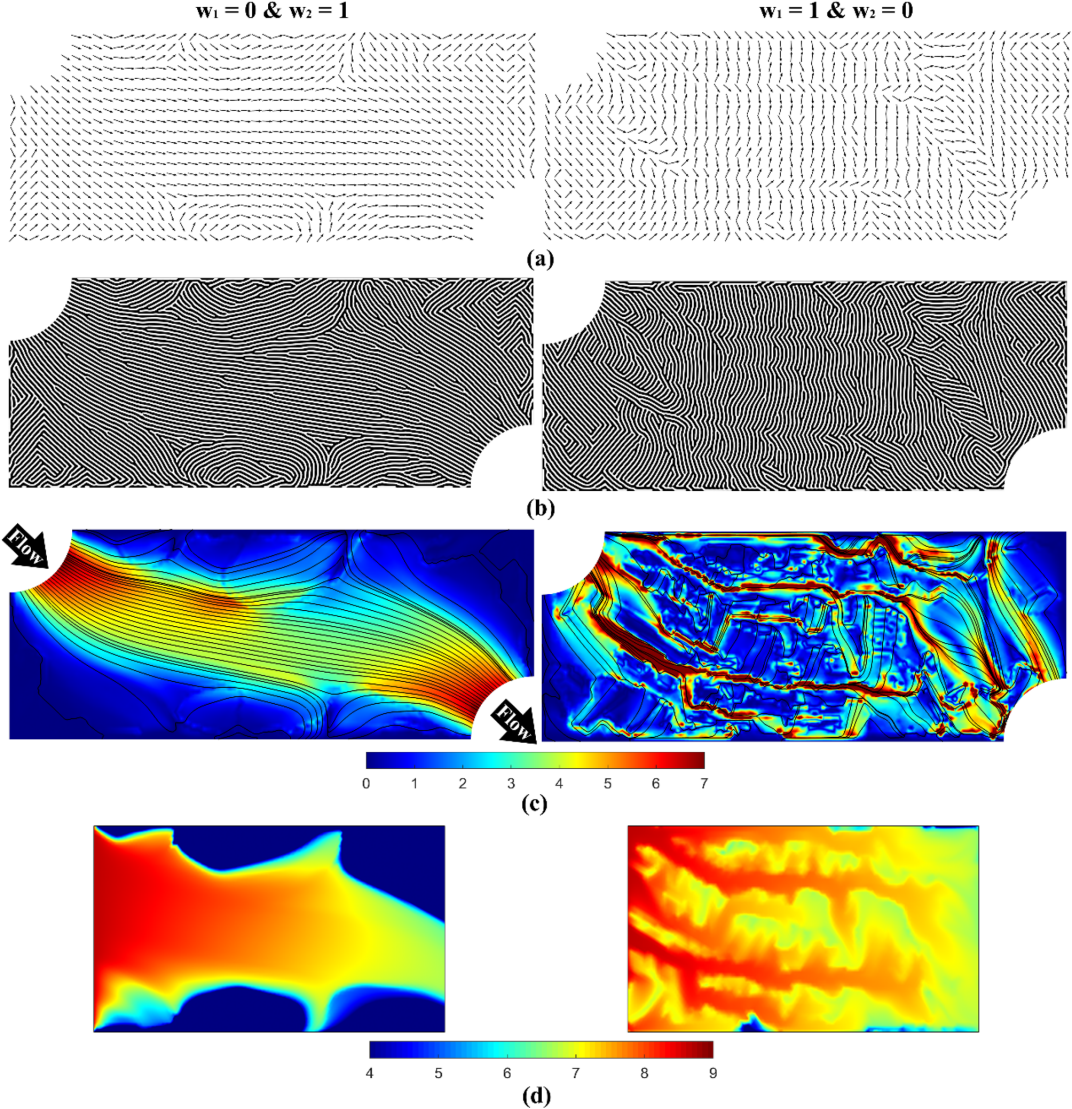
Single-objective optimization results for a representative microreactor design problem. In each image the fluid inlet is in the upper left and the outlet is in the lower right. (**a**) Optimized orientation fields from the homogenization-based optimization. (**b**) Diffusion-based dehomogenized microreactor flow channels. (**c**) Velocity field (units: m/s). (**d**) Reactant concentration field (units: mol/m^3^). Left column: the flow resistance objective was minimized; Right column: the reactant distribution variability objective was minimized.

The limits of the weighting interval represent a single objective optimization problem as one of the weights becomes zero. Figure 2a,b, respectively, reveal the optimized orientation fields and resultant dehomogenized microchannel designs at the limits of our designated design space, where only one objective is solved for. In the case where only the flow resistance is minimized, clear parallel channel flow paths connecting the inlet region to the outlet region emerge, left image in Fig. 2b, with minimal flow under walls or ribs into the underlying microreactor porous gas diffusion layer. In contrast, when only the reactant distribution variability is minimized, vertical channel flow paths emerge to impede the natural flow of the fluid and disperse it into the gas diffusion layer, right image in Fig. 2b, where the reaction would ultimately occur. Figure 2c,d illustrate the corresponding velocity and reactant concentration fields, respectively, for the corresponding single objective designs. When the flow resistance is minimized, a velocity field with smooth streamlines emerges, but at the expense of a non-uniform reactant concentration field. Conversely, when the reactant distribution variability is minimized, a chaotic velocity field emerges to permit a more uniform reactant concentration field, but logically at the expense of a higher fluid flow resistance.

**Figure 3:**
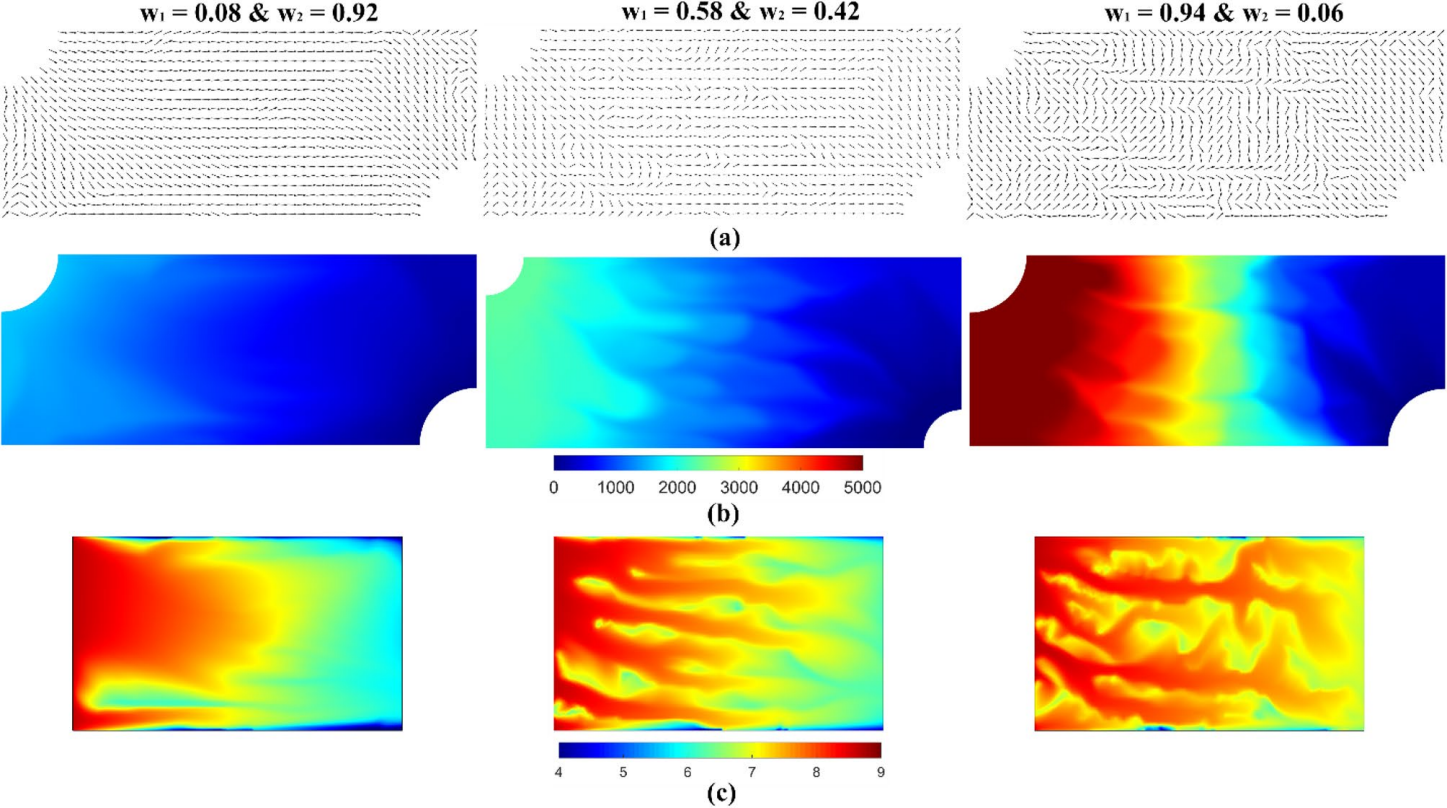
Results of the homogenization-based optimization routine for the three different weighting schemes in the Pareto set. Each column represents a different combination of the objective function weights. (**a**) Optimized orientation field. (**b**) Pressure field (units: Pa). (**c**) Reactant concentration field (units: mol/m^3^).

For a deeper understanding of how the flow field transforms throughout the Pareto set, three designs were selected and presented in Fig. 3. The optimized orientation field, pressure field, and reactant concentration field for the selected designs are presented in Fig. 3. As the reactant distribution variability weight (${w}_{1}$) increases, the reactant concentration distribution becomes more uniform, as shown in Fig. 3c. However, Fig. 3b reveals that reactant distribution uniformity comes at the cost of a significant increase in the pressure drop. The solution that appropriately balances these two competing design objectives uses a balanced weighting scheme, ${w}_{1}=0.58$ and ${w}_{2}=0.42$, which maintains a relatively low pressure drop while yielding a more uniform reactant distribution.

**Figure 4:**
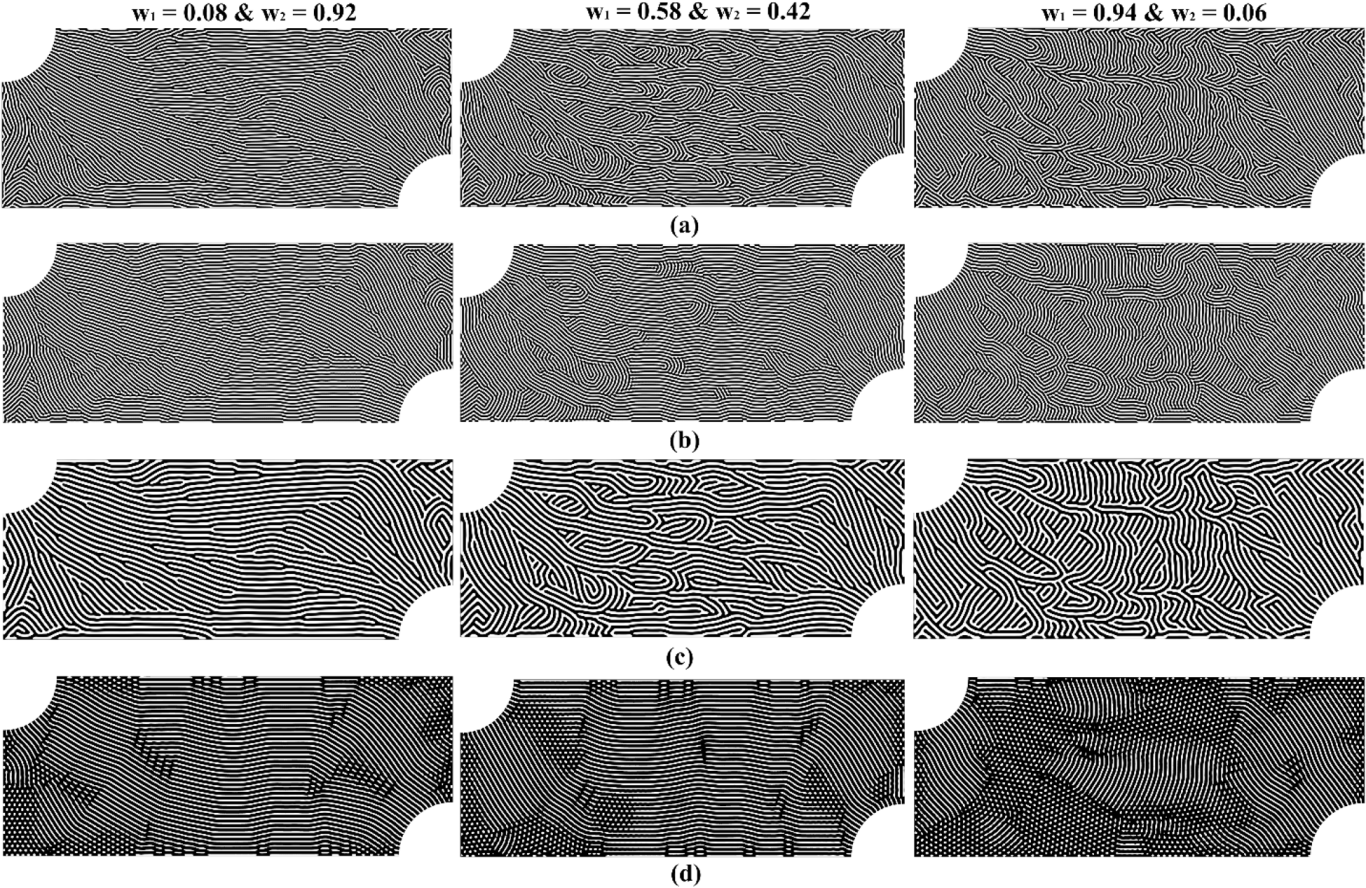
Diffusion-based dehomogenized microreactor flow channel designs for the three different weighting schemes in the Pareto set. Each column represents a different combination of the objective function weights. Each row represents a different design type defined by the Swift-Hohenberg model parameter settings. (**a**) “Balanced” design. (**b**) “Parallel” design. (**c**) “Wide” design. (**d**) “Semi-discrete” design.

The steady-state equation-based dehomogenization technique was used to convert the selected optimized orientation fields into distinct microchannel designs. Figure 4a presents the microchannel structures for design described in Fig. 3. A comparison of the three designs reveals that as the reactant distribution variability weight ($${w}_{1})$$ increases, more fluid flow channels emerge perpendicular to the primary inlet-to-outlet flow path to encourage a greater dispersion of the reactant fluid into the underlying gas diffusion layer of the microreactor.

### Rapid generative design expansion

A steady-state equation-based dehomogenization technique was used to convert the selected optimized orientation fields into distinct microchannel designs. Figure 4a presents the microchannel structures using the “balanced” dehomogenization setting (Table 1) for design described in Fig. 3. A comparison of the three designs reveals that as the reactant distribution variability weight ($${w}_{1})$$) increases, more fluid flow channels emerge perpendicular to the primary inlet-to-outlet flow path to encourage a greater dispersion of the reactant fluid into the underlying gas diffusion layer of the microreactor.

**Table 1:** Swift-Hohenberg model parameter settings for different types of designs.

Design type	Swift-Hohenberg model parameter settings			
	$w$	$\alpha$	$\varepsilon$	$g$
Balanced	0.9	0.7	1	0
Parallel	0.9	0.2	1	0
Wide	1.5	0.7	1	0
Semi-discrete	1.25	0.1	0.01	0.99

Due to the rapid dehomogenization speed of the proposed steady-state model, a generative design approach could be deployed to greatly expand the number of flow channel designs. The parameters in the Swift-Hohenberg model were adjusted, per Table 1, to permit the generation of three additional and distinctly different microchannel geometries for each optimized orientation field in the grid search.

First, “parallel” designs were established by reducing an anisotropic parameter, $\alpha$ [refer to Eq. (8) in Methods], to soften the orientation requirement and encourage flow fields with primarily parallel channels. Figure 4b illustrates the dehomogenized “parallel” designs for the three different weighting schemes. The resultant flow fields favor parallel channels, but at the expense of smoothing out some of the details found in the “balanced” design configurations. Next, “wide” designs were established by increasing the channel width parameter, $w$ (refer to Eqs. 7 and 8 in Methods), to foster designs at a larger length scale. Figure 4c reveals the dehomogenized “wide” designs for the three different weighting schemes. The resultant flow fields respect the optimized orientation while introducing the potential of larger channel geometries into the design space. Finally, a “semi-discrete” design was established by adjusting the pattern-type control parameters, $\varepsilon \text{ and } g$ [refer to Eq. (4) in Methods], and reducing the anisotropic parameter, $\alpha$, to encourage the generation of discrete microstructures. Figure 4d reveals the dehomogenized “semi-discrete” designs for the three different weighting schemes. The resultant flow fields appear to favor discrete features in locations where the orientation is not ideal for easily producing parallel channels. For demonstration purposes, only three additional designs were generated here for each orientation field. The number of generative designs can be efficiently further expanded using the proposed rapid steady-state dehomogenization technique with the caveat that the multiphysics performance of expanded generative designs should be further validated to confirm consistency with homogenization-based optimization assumptions. For example, in the “parallel” case, the optimized orientation is not strictly consistent with the optimization result after dehomogenization. In the “wide” and “semi-discrete” cases, the assumed unit cell geometry is not precisely recovered after dehomogenization.

**Figure 5:**
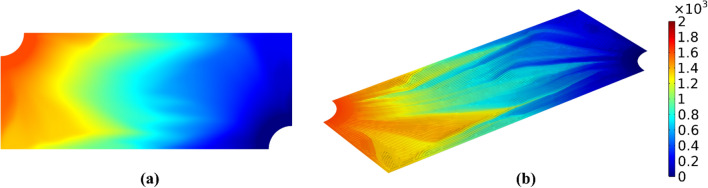
Pressure distribution for the “balanced” design (w_1_ = 0.08 & w_2_ = 0.92) (units: Pa). (**a**) 2D anisotropic porous media. (**b**) 3D dehomogenized microchannels including the porous gas diffusion layer; refer to Fig. 8.

It is noted that the performance mismatch before and after the dehomogenization step is still an open question across many disciplines due to the unpredictable nature of local features after dehomogenization. For solid elastic structures, the structural stiffness mismatch caused by discontinuous load transfer has been reported^34,35^. For flow-driven structures, the pressure and velocity mismatch due to undesired local branching, recombining, and dead ends has also been recently reported^11,25^. While the “balanced” design type in Table 1 is intended to closely match the homogenization-based performance, it should be acknowledged that additional geometric fine tuning is needed in order to mostly recover the optimized performance. To demonstrate the agreement between the predicted 2D homogenized response and the dehomogenized 3D response, Fig. 5 shows both pressure distributions for the “balanced” design (w_1_ = 0.08 & w_2_ = 0.92). While both their global trends and magnitudes generally agree well, local disagreements are still present. Solving any performance mismatch issue in detail is less of a focus for this work since our primary motivation is to explore the generative design concept by rapidly producing many “near optimal” designs. Nonetheless, such in depth validation can be performed, and the reader is referred to^30^ for a representative study.

### Multi-region microreactor designs

**Figure 6:**
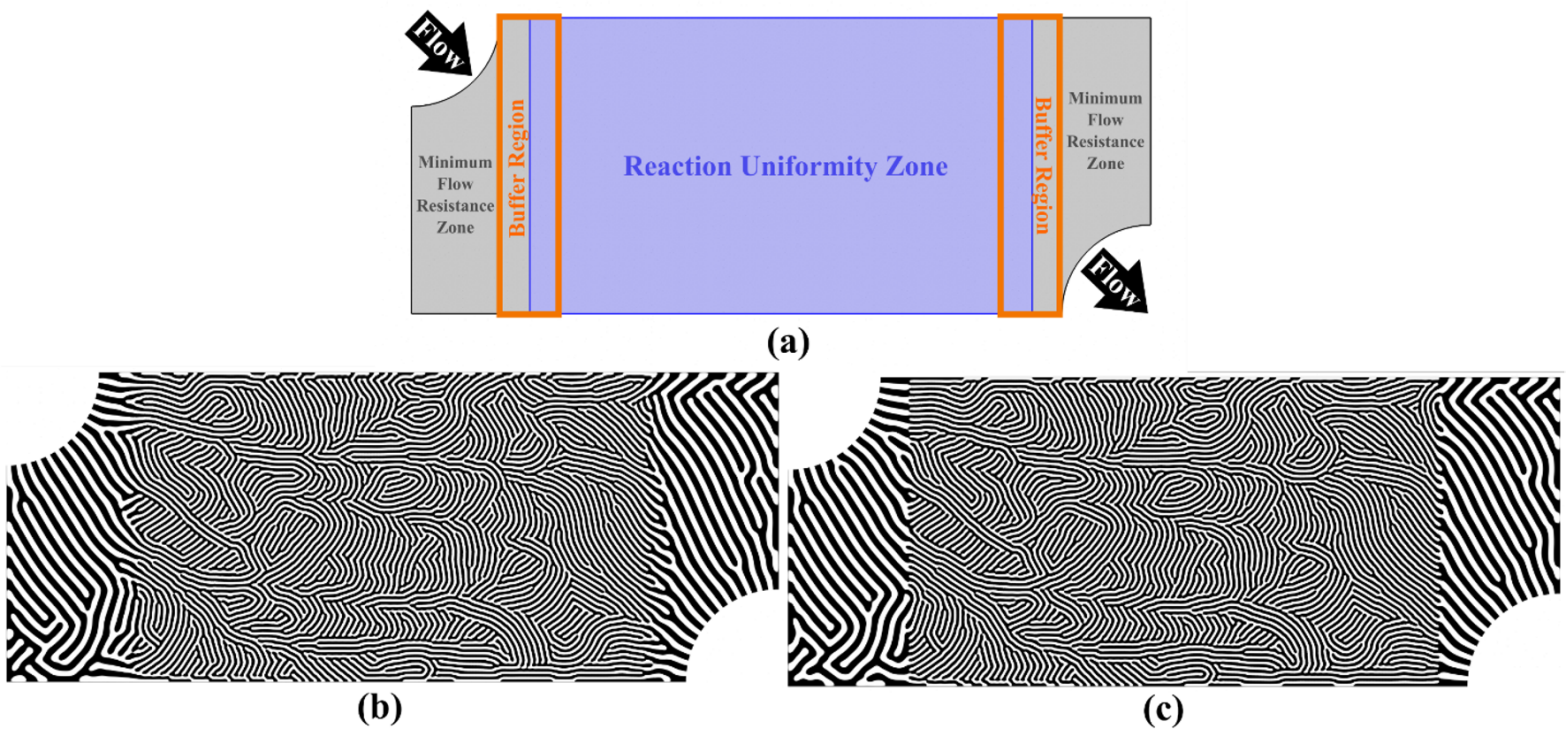
Multi-region microreactor flow field designs. (**a**) Design domain with zones identified based on different physical objectives. (**b**) Diffusion-based dehomogenized design including the buffer region. (**c**) Diffusion-based dehomogenized design without the buffer region.

During the diffusion-based dehomogenization process, the pattern or structure designer has an added layer of control and flexibility that may be exploited to create unique multi-region microreactors with functionally graded channel geometries. For example, Fig. 6a illustrates a design domain where zones have been identified based on different physical objectives. To address the localized requirements, a spatially varying channel width parameter can be defined to promote wider channels in the minimum flow resistance zone and narrower channels in the reaction uniformity zone. In addition, buffer regions can be introduced to ensure a gradual transition between discrete design features. Figure 6b,c illustrates the multi-region dehomogenized flow channel designs with and without the buffer region, respectively. It is worth noting that the feature transition is seamless between the neighboring zones, even if the buffer region is not included. This characteristic is unique to the Swift-Hohenberg model and can be challenging to achieve without a diffusion-based technique, particularly when there is a hard boundary between zones.

**Figure 7:**
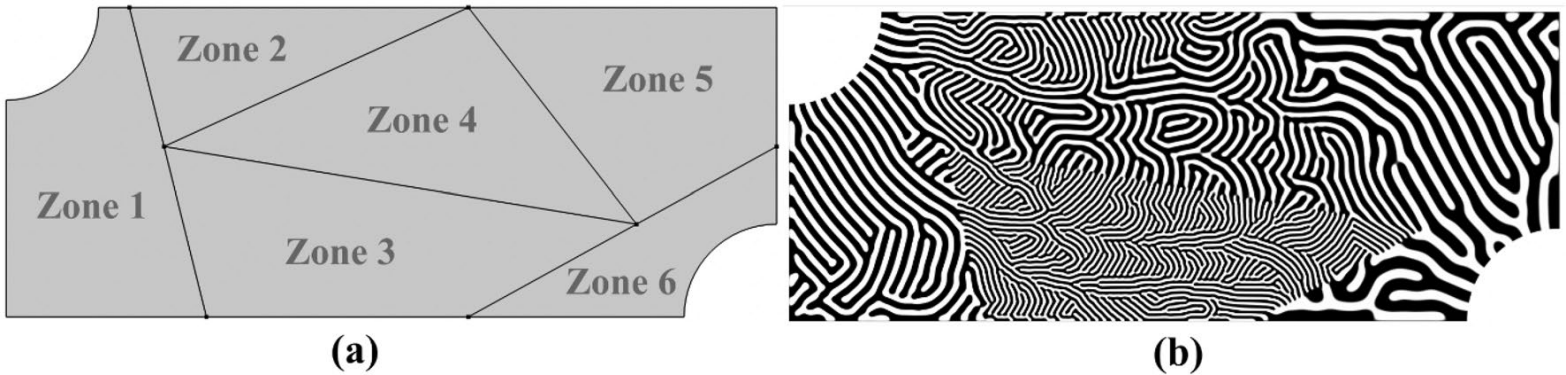
Microreactor design domain with subdomains of arbitrary shapes and sizes. (**a**) Zone partitioning. (**b**) Diffusion-based dehomogenized multi-region flow channel design with spatially varying channel widths.

To further highlight the versatility of the approach, Fig. 7 illustrates a unique six-zone microreactor with subdomains that vary in shape, size, and channel width. This type of architecture could prove to be useful in lab-on-a-chip applications where different channel designs and scales are required to meet separate designated physical objectives^36,37^. For example, the flow field could be constructed such that “Zone 1” is the inlet region, “Zone 2” and “Zone 3” are mixing regions, “Zone 4” is the reaction region, “Zone 5” is the drainage region, and “Zone 6” is the outlet region. Due to the computational efficiency of the steady-state dehomogenization process, a generative design approach could be implemented to explore the vastness of the multi-region design domain for an array of applications. It should be noted that both the zone partitioning, and the spatially varying design features can also be fine-tuned within an additional corresponding optimization representation.

## Discussion

In total, 200 microreactor flow channel designs were created for our multiphysics design problem using the proposed steady-state dehomogenization technique. The solutions spanned the full range of objective function weights defined by a grid search, in addition to the four distinct categories of design features controlled by the parameter settings, Table 1, in the Swift-Hohenberg model. For each design category, 50 structures were generated representing every sampled point in the grid search. The average time required to produce a single dehomogenized flow field design was 119 s for the “balanced” setting, 70 s for the “parallel” setting, 124 s for the “wide” setting, and 81 s for the “semi-discrete” setting, as listed in Table 1. All of the results from the computational experiments that are reported in this article were performed on a desktop computer with a Xeon Gold 6230 CPU (2.1 GHz) and 384 GB memory. It should also be noted that the reported computational time is based on a COMSOL and MATLAB implementation. Timing studies are logically dependent on the selection of the solver, software, programing language, and computational hardware. In general, larger values of the anisotropic parameter ($\alpha$) required slightly longer computational time due to the heightened orientation requirement that had to be satisfied in the final design. Altogether, 200 unique and distinctly different microreactor flow channel designs were created in just over 5.5 h, rendering the steady-state dehomogenization technique a viable tool in generative design applications. The supplementary material contains all 200 individual microchannel flow field designs that were created and animations that reveal how the flow fields evolve as the weighting scheme in the objective function changes.

## Methods

### Multi-objective optimization

**Figure 8:**
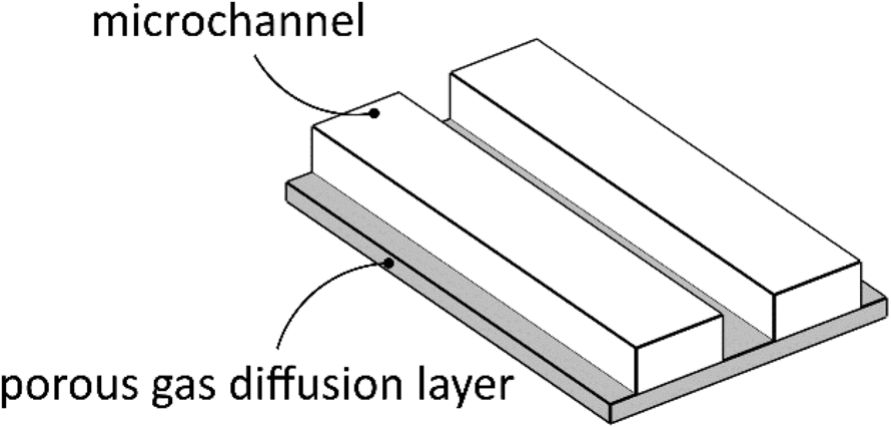
Microreactor unit cell configuration with microchannels and a thin, porous gas diffusion layer.

The multi-objective optimization problem of minimizing fluid flow resistance (i.e., pressure drop) and reactant distribution variability, common in the design of microreactors^38^, was considered in this study. A gradient-based anisotropic porous media optimization method, as presented in the work of Zhou et al.^25^, was employed to generate optimized orientation fields for equivalent porous materials. A unit cell microstructure is adopted assuming microchannels positioned on top of a thin, porous gas diffusion layer, as illustrated in Fig. 8. Each optimized orientation field was then converted into a microreactor flow channel design using the proposed steady-state dehomogenization process.

The method proceeds by optimizing the material orientation at each point in space by mapping the design variables to an orientation tensor. Next, an anisotropic porous medium permeability tensor is rotated according to the local material orientation. The unidirectional anisotropic porous medium contains two effective permeabilities along and orthogonal to the channel direction. In the case of the specific unit cell dimensions reported in^25^, they are $8.65\times {10}^{-9} {\mathrm{m}}^{2}$ and $2.33\times {10}^{-11} {\mathrm{m}}^{2}$, respectively. Finally, laminar fluid flow through the designed equivalent porous media is analyzed using the Stokes equation, Darcy’s law, and the advection–diffusion-reaction equation. The design variables are updated based on the sensitivity analysis of the objective function, which is given by the following,

1 $$\begin{array}{c}F={w}_{1}{f}_{1}/{f}_{1}^{\left(0\right)}+{w}_{2}{f}_{2}/{f}_{2}^{\left(0\right)},\end{array}$$

2 $$\begin{array}{c}{f}_{1}={\int }_{{\Omega }_{r}}{\left(\frac{c-{c}_{avg}}{{c}_{avg}}\right)}^{2}d{\Omega }_{r},\end{array}$$

3 $$\begin{array}{c}{f}_{2}=\frac{1}{2}{\int }_{\Omega }\nabla \mathbf{v}\cdot \left(\nabla \mathbf{v}+{\left(\nabla \mathbf{v}\right)}^{\mathrm{T}}\right)d\Omega .\end{array}$$

**Figure 9:**
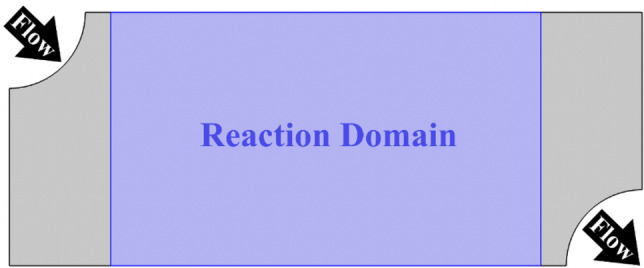
Microreactor design domain, $\Omega$. The reaction domain, $\mathrm{\Omega r}$, is highlighted in blue. The inlet is located at the top left corner and the outlet is located at the bottom right corner.

The objective function, $F$, in Eq. (1) contains a weighted sum of two design requirements, given by the average variation of reactant concentration, ${f}_{1}$, defined in Eq. (2), and the flow resistance, ${f}_{2}$, defined in Eq. (3). Both terms are normalized by their initial values at the first optimization iteration ${f}_{1}^{\left(0\right)}$ and ${f}_{2}^{\left(0\right)}$. Again, the weights, ${w}_{1}$ and ${w}_{2}$, control how much the optimization favors designs with a more uniform reactant distribution versus designs with a lower flow resistance, respectively. Within Eqs. 2 and 3, $c$ represents the reactant concentration field; $\mathbf{v}$ represents the velocity field. As illustrated in Fig. 9, ${\Omega }_{r}$ represents the prescribed reaction domain and $\Omega$ represents the entire design domain. The design domain consists of a fluid inlet region in the upper left-hand corner and an outlet region in the bottom right-hand corner. Refer to^25^ for greater details about dimensions and physical properties.

COMSOL and MATLAB were used to solve the multiphysics and multi-objective optimization problem, for which automatic sensitivity analysis was performed using the “sensitivity” module in the COMSOL “mathematics” interface. Finally, the optimization routine was performed using the method of moving asymptotes optimizer^39^. Although not the primary focus of this article, further extensive details about the homogenization-based anisotropic porous media optimization approach for this problem setup are available in^25^.

### Steady-state dehomogenization

Bioinspired patterns were used to dehomogenize the optimized orientation field into microreactor flow channel designs via the Swift-Hohenberg model. The Swift-Hohenberg model was originally established to study the Rayleigh-Bénard system where convective instability causes rolls and hexagon patterns to emerge^33,40,41^. The system is given by the single equation,

4 $$\begin{array}{c}\frac{\partial u}{\partial t}=-{\left({\nabla }^{2}+{k}^{2}\right)}^{2}u+\varepsilon u+g{u}^{2}-{u}^{3}-2{q}^{2}\nabla \cdot \left({\varvec{D}}\nabla u\right).\end{array}$$

The last term in Eq. (4) was introduced to the model, in^27^, as a production gradient to permit anisotropic diffusion such that the patterns evolve according to a prescribed orientation field. The anisotropic diffusion tensor (${\varvec{D}}$) was defined using a normalized orientation vector, ${\varvec{p}}=({p}_{1},{p}_{2})$, as follows,

5 $$\begin{array}{c}{\boldsymbol{\varphi }}_{\perp }=\left[\begin{array}{cc}\mathrm{cos}\theta & -\mathrm{sin}\theta \\ \mathrm{sin}\theta & \mathrm{cos}\theta \end{array}\right]\left[\begin{array}{c}{p}_{1}\\ {p}_{2}\end{array}\right]=\left[\begin{array}{cc}0& -1\\ 1& 0\end{array}\right]\left[\begin{array}{c}{p}_{1}\\ {p}_{2}\end{array}\right]=\left[\begin{array}{c}{-p}_{2}\\ {p}_{1}\end{array}\right],\end{array}$$

6 $$\begin{array}{*{20}l} {\user2{D}\left( {\user2{\bar{\varphi }}} \right) = \user2{\bar{\varphi }}_{ \bot } \otimes ~\user2{\bar{\varphi }}_{ \bot } = \left[ {\begin{array}{*{20}c} {\bar{p}_{2} \bar{p}_{2} } & { - \bar{p}_{1} \bar{p}_{2} } \\ {sym.} & {\bar{p}_{1} \bar{p}_{1} } \\ \end{array} } \right],} \\ \end{array}$$

where ${\overline{\boldsymbol{\varphi }} }_{\perp }$ represents the *optimized* orientation vector that has been rotated 90°. The vector must be rotated 90° so that the major axis of a striped pattern is aligned with the primary axis of the flow field to create distinct microchannels during the dehomogenization process^25,27^. This is due to an underlying feature of the Swift-Hohenberg model which orients patterns perpendicular to the production gradient^31^.

**Figure 10:**
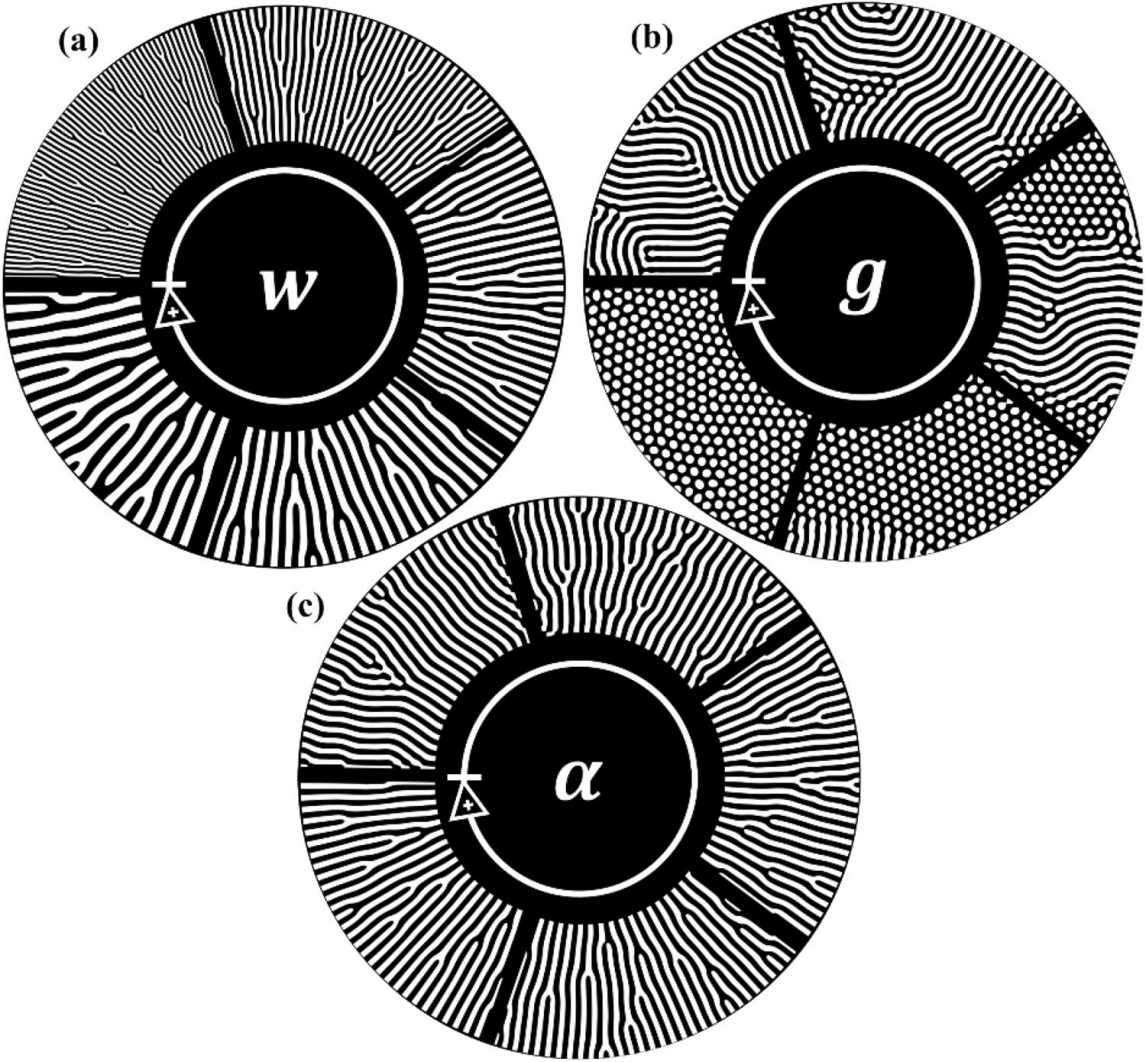
Parameter adjustment in the Swift-Hohenberg model. The arrow in each sub-figure indicates the direction of increase for the parameter value. (**a**) Channel width parameter, w (prescribed radial orientation). (**b**) Striped or spotted pattern control parameter, g (ε is held constant). (**c**) Anisotropic control parameter, α (prescribed radial orientation).

Various geometric features of the microreactor flow channels can be introduced using additional parameters presented in the Swift-Hohenberg model. The parameters, $k$ and $q$, in Eq. (4) were defined as follows,

7 $$\begin{array}{c}k=\sqrt{\frac{{\pi }^{2}}{{w}^{2}}-{q}^{2}},\end{array}$$

8 $$\begin{array}{c}q=\frac{\alpha \pi }{w},\end{array}$$

such that the parameter, $w$, controls the frequency of the pattern. In the case of striped patterns, larger values of $w$ correspond to wider channels while smaller values correspond to narrower channels, as described in^27^ and illustrated in Fig. 10a. The constants, $\varepsilon$ and $g$, in Eq. (4) control whether a striped or spotted bioinspired pattern emerges, as shown in Fig. 10b, where $\varepsilon$ is held constant and $g$ is varied (since $g>0$ drives the development of spotted patterns)^27^. In Eq. (8), the parameter, $\alpha$, controls the level of anisotropy which defines how tightly the patterns must adhere to the prescribed orientation field. For uniformly spaced striped patterns, higher anisotropic values correspond to designs with more branching to respect the prescribed orientation field, while lower anisotropic values correspond to designs with more parallel channels, as illustrated in Fig. 10c.

Due to the transient quality of pattern development in nature, the Swift-Hohenberg model is often solved in time to capture the temporal dynamics of the system^27,40,42–45^. However, when applied as a dehomogenization technique to engineering structural design applications, the details on exactly how the pattern evolves throughout time are not a priority. Therefore, when used for this application, the time evolution process can be skipped by solving the steady-state equation. Compared to other pattern generation models (e.g., the Brusselator model^46^, the Schnackenberg model^47^, and the Gray–Scott model^48^) the Swift-Hohenberg model is unique, because it generates patterns from a single variable equation instead of a system of coupled equations. Despite this distinctive attribute, steady-state solution demonstrations have been predominantly limited to numerical exercises^49–51^.

Here, the partial differential equation was discretized to solve the steady-state Swift-Hohenberg equation by assigning,

9 $$\begin{array}{c}\frac{\partial u}{\partial t}=0.\end{array}$$

**Figure 11:**
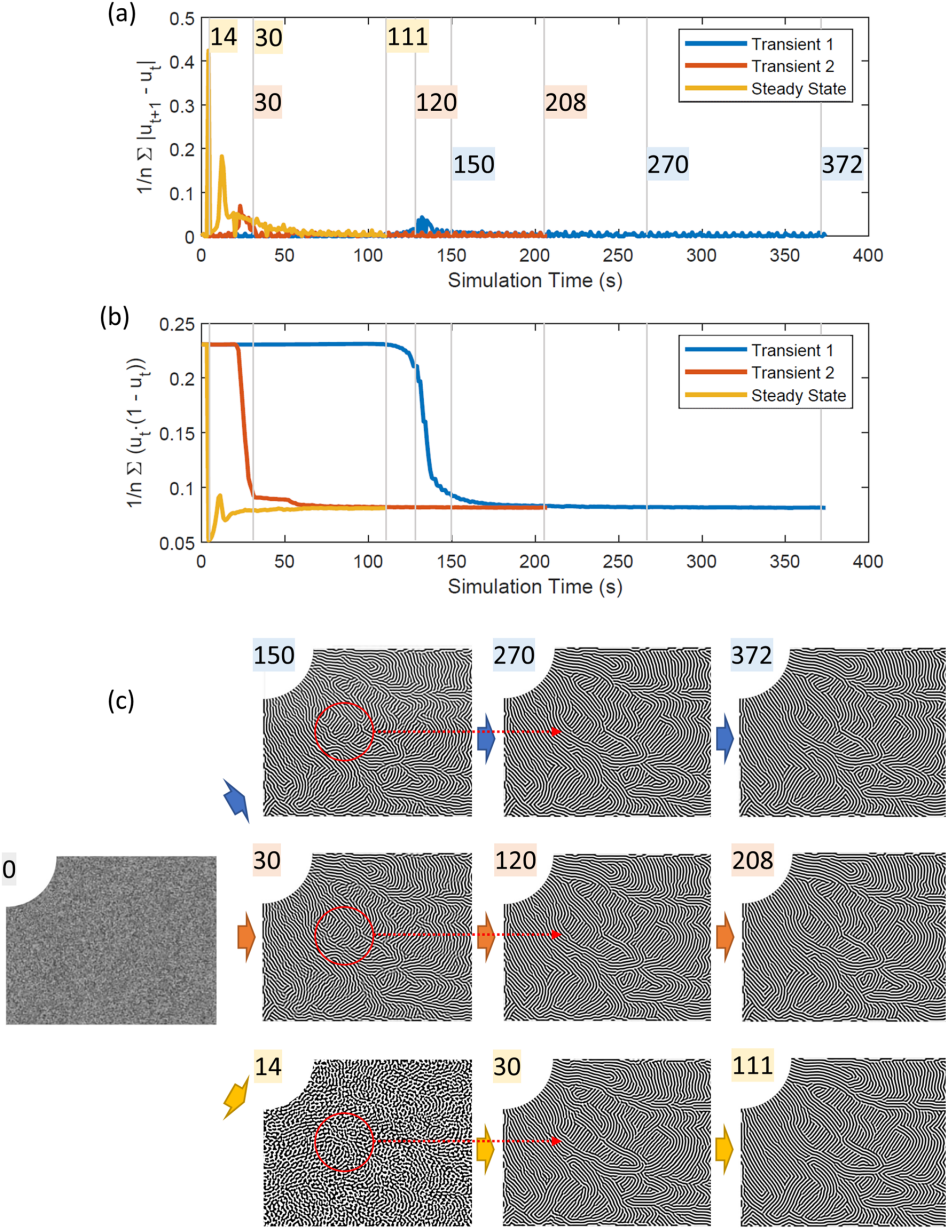
Comparison of convergence criteria with respect to simulation time for one steady-state and two transient dehomogenization implementations. (**a**) The average change in state variable, u; note that the overlaid time stamps correspond to the images in the (**c**) subfigure. (**b**) Amount of non-binary elements on the domain. (**c**) Select design images (showing the left half of the full domain for clarity) at different times; note that the Transient 1, Transient 2, and Steady State design images are shown in the top, middle, and bottom rows, respectively. The red circles highlight a representative region where design changes are observed from the earlier time snapshot to the next.

A stationary solver was used in combination with the Newton–Raphson method for the nonlinear component of the equation. The nondimensionalized design domain was discretized into ~ 60 k free triangular elements (in two dimensions) with initial conditions given by, ${u}_{0}\approx \sqrt{\varepsilon }$. There are two key benefits of solving a steady-state pattern generation model over a transient model. First, the steady-state model guarantees convergence of the solution. This is because the solution must satisfy Eq. (9) which states that the change in concentration with respect to the change in time is zero. Second, the steady-state model generally solves faster without the need to extensively optimize solver parameters. To demonstrate this, a set of both steady-state and transient simulations are performed. Their convergence properties are presented in Fig. 11. The transient models were run until t = 1 s using two time stepping techniques. The first transient implementation, Transient 1 in Fig. 11, used an implicit backward differentiation formula (BDF) time stepping strategy with default adaptive steps from the commercial software. The second transient implementation, Transient 2 in Fig. 11, used the same BDF strategy with a fixed time step of 0.005 s. It is observed that transient solutions can behave differently based on solver parameter settings. The steady-state model was run until the relative solver convergence tolerance was below 0.1. The average change in the state variable, *u*, is shown in Fig. 11a, where the steady-state model initially experiences large changes in the state variable before fine tuning the design with relatively smaller changes. By comparison, both transient simulations begin with smaller changes in *u* before experiencing a large change in state, then smaller changes to convergence. The average amount of non-binary states, relating to the convergence of the solution, for each simulation is shown in Fig. 11b. Similar trends are observed where the steady-state solver reaches a nearly converged solution much faster than the transient solvers. Note that the transient solvers may possibly be heuristically adjusted through a trial-and-error approach to achieve comparable convergence performance. However, the convergence criteria for steady-state solvers can be set more straightforwardly with minimal manual adjustment required. A series of time stamped design images, focusing on the left half of the design domain for clarity, from each simulation are provided in Fig. 11c to support visual comparison. The red circled regions in Fig. 11c highlight representative portions of the design where changes are observable from the earlier time snapshot to the next.

**Figure 12:**
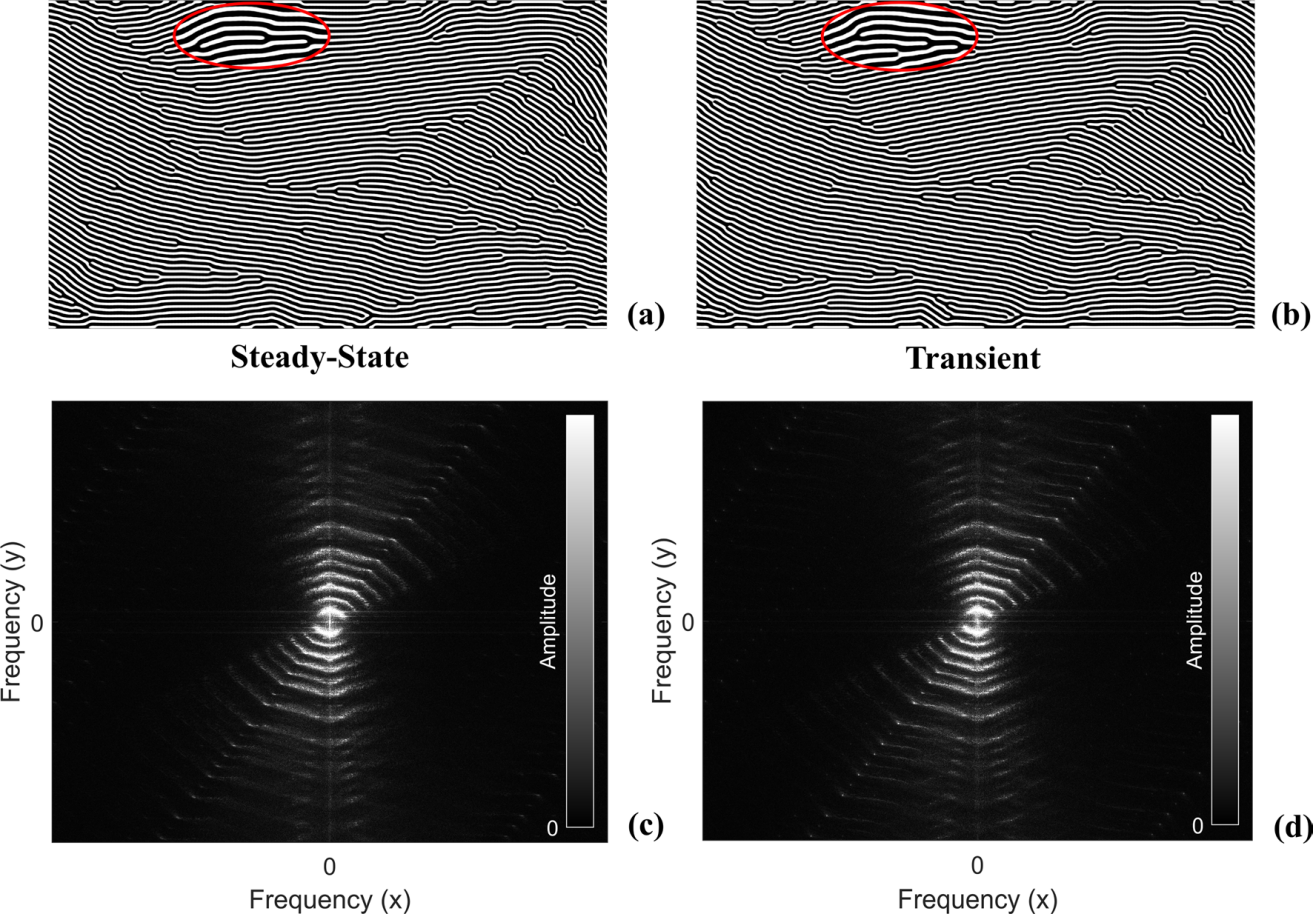
Comparison of steady-state versus transient Swift-Hohenberg model. (**a**) Steady-state spatial domain with magnified region highlighted by a red oval. (**b**) Transient spatial domain with magnified region highlighted by a red oval. (**c**) Steady-state frequency domain. (**d**) Transient frequency domain.

Furthermore, the steady-state solver produces structurally similar designs to those produced with the transient solver. Consider the two fully converged designs shown in Fig. 12. The final solutions generated in the spatial domain maintained structural similarities but with slightly different branching locations, as highlighted by the magnified regions in Fig. 12a,b. Next, a two-dimensional Fourier transform analysis was adopted to verify the statistical similarity between the spatial patterns produced by each model. Figure 12c,d shows the steady-state and transient solutions in the frequency domain, respectively. A pixel-wise average difference of amplitudes between the two images was computed to reveal a 3.6% difference. This confirms the structural similarity achieved using the steady-state solution, and justifies the interchangeable use of models for engineering design. Therefore, the steady-state Swift-Hohenberg model may be applied to the rapid dehomogenization process with confidence that the proper solutions are being generated.

## Conclusion

In this paper, the steady-state Swift-Hohenberg model was proposed as a rapid diffusion-based dehomogenization technique for a multiphysics, fluid flow-based, microreactor application. Bioinspired diffusion-based pattern generation models yield designs that are geometrically distinct and can be formed in complex design domains, while maintaining the seamless transition between structural features (e.g., orientation and channel dimension). However, these models are often computationally expensive to solve, as they are conventionally represented in time and space. By utilizing a steady-state equation, we removed the time dependency, which resulted in a simpler solver setup and potential computational speed-up. As a result, this work presents a diffusion-based dehomogenization tool that can be practically implemented to enable generative design for structural engineering applications. To highlight the feasibility and uniqueness of the proposed method, the dehomogenization tool was used to explore the design space of a multi-objective optimization problem for microreactor flow channels. Altogether, 200 unique and distinctly different designs were generated to illustrate the optimization and parameter space that becomes easily accessible when implementing this unique form of the Swift-Hohenberg model. The diffusion-based dehomogenization tool was further extended to the design of multi-region microreactor flow channels. The capability of spatially varying design parameters presents an added layer of fluid flow control that can be exploited to adjust the functionality of different microreactor subdomains to meet specific physical objectives. For future work, we propose applying the rapid steady-state dehomogenization technique to other multiphysics engineering design applications, e.g., thermal-fluid systems.

### Supplementary Information

Supplementary Video 1.

Supplementary Information 1.

## Data Availability

The supplementary material contains all 200 individual microchannel flow field designs that were created, and animations that reveal how the flow fields evolve as the weighting scheme in the objective function changes. Further details are available upon request from the corresponding author.
