# Supplementary material for: Generative design of large-scale fluid flow structures via steady-state diffusion-based dehomogenization
Source: Sci Rep. 2023 Sep 1;13:14344. doi: 10.1038/s41598-023-41316-w (PMC10474040; doi:10.1038/s41598-023-41316-w)
Supplement: Supplementary file 2 — Supplementary Information 1. [file 41598_2023_41316_MOESM2_ESM.docx]

**Supplementary A: Diffusion-based dehomogenization of “balanced” design type.**

**
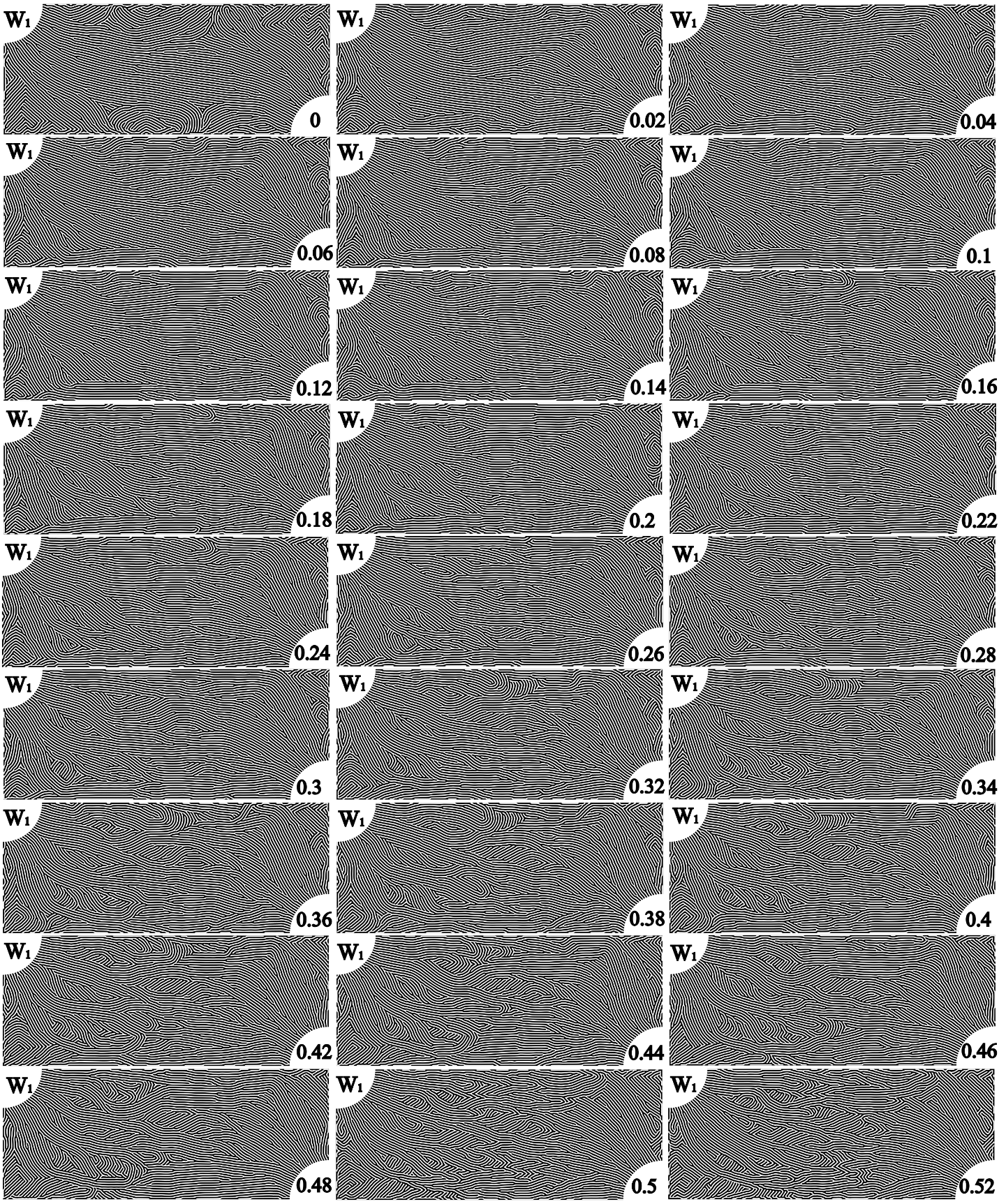
**

**Supplementary A: Continued.**


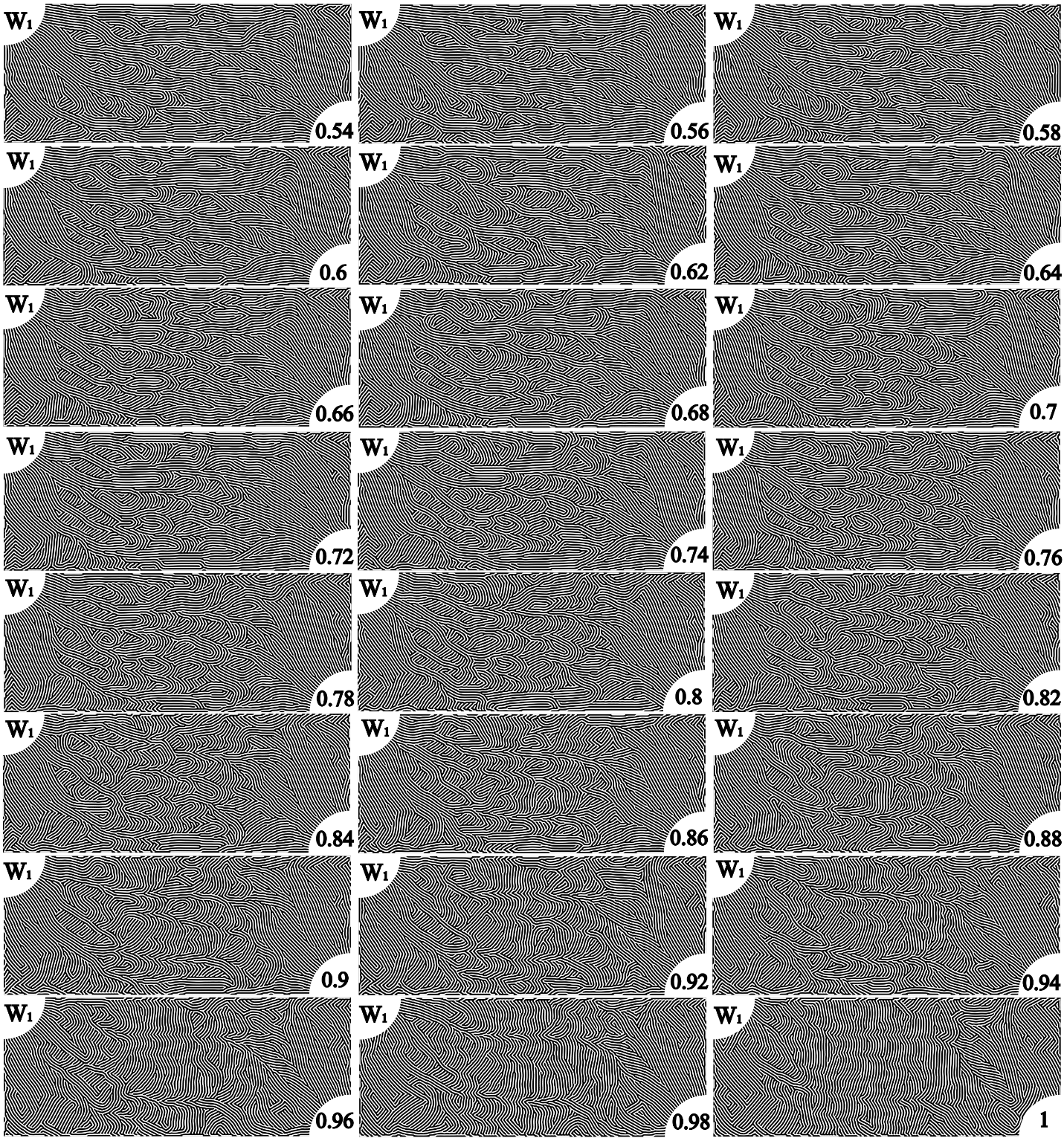


**Supplementary B: Diffusion-based dehomogenization of “parallel” design type.**


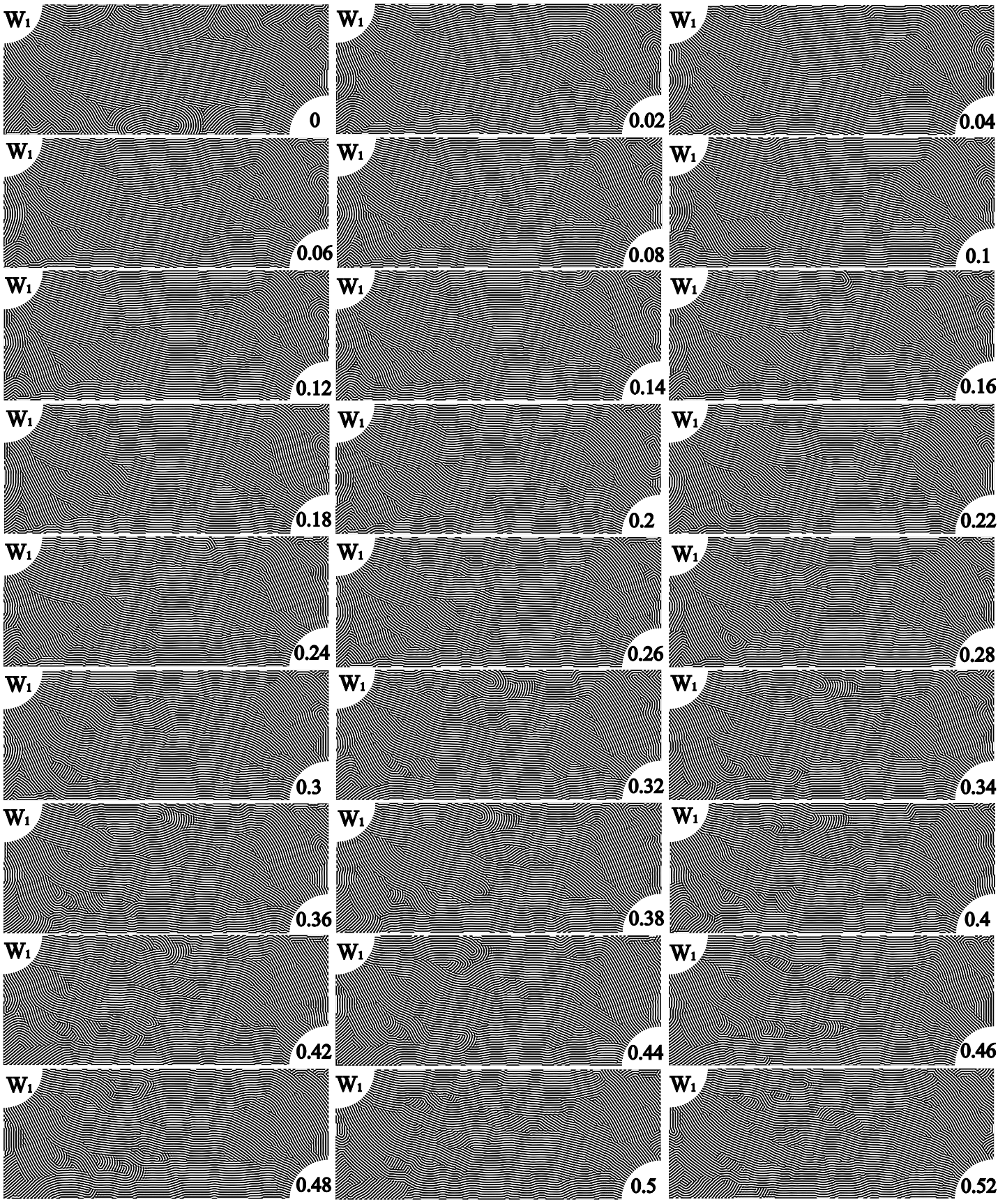


**Supplementary B: Continued.**


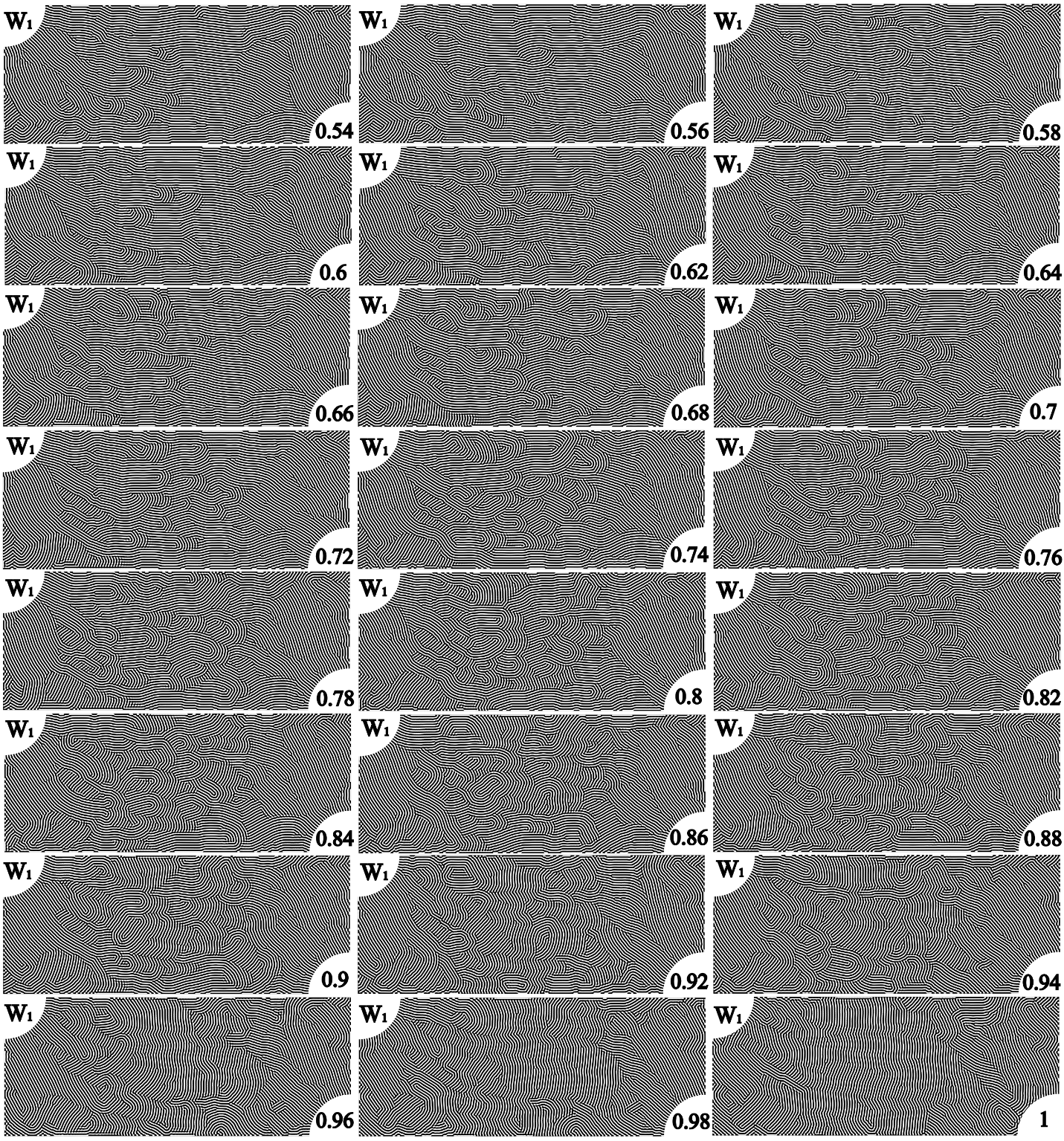


**Supplementary C: Diffusion-based dehomogenization of “wide” design type.**

**
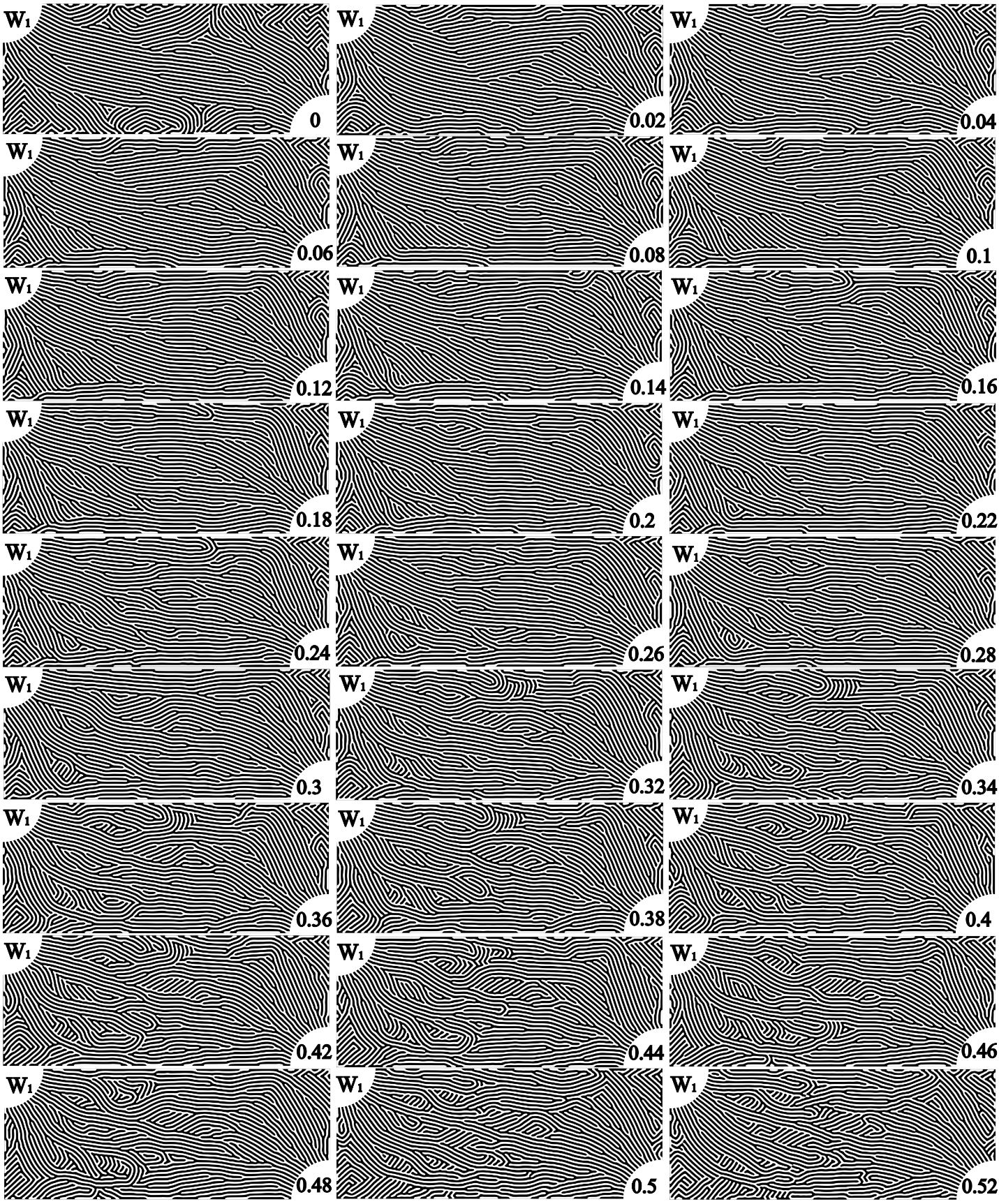
**

**Supplementary C: Continued.**

**
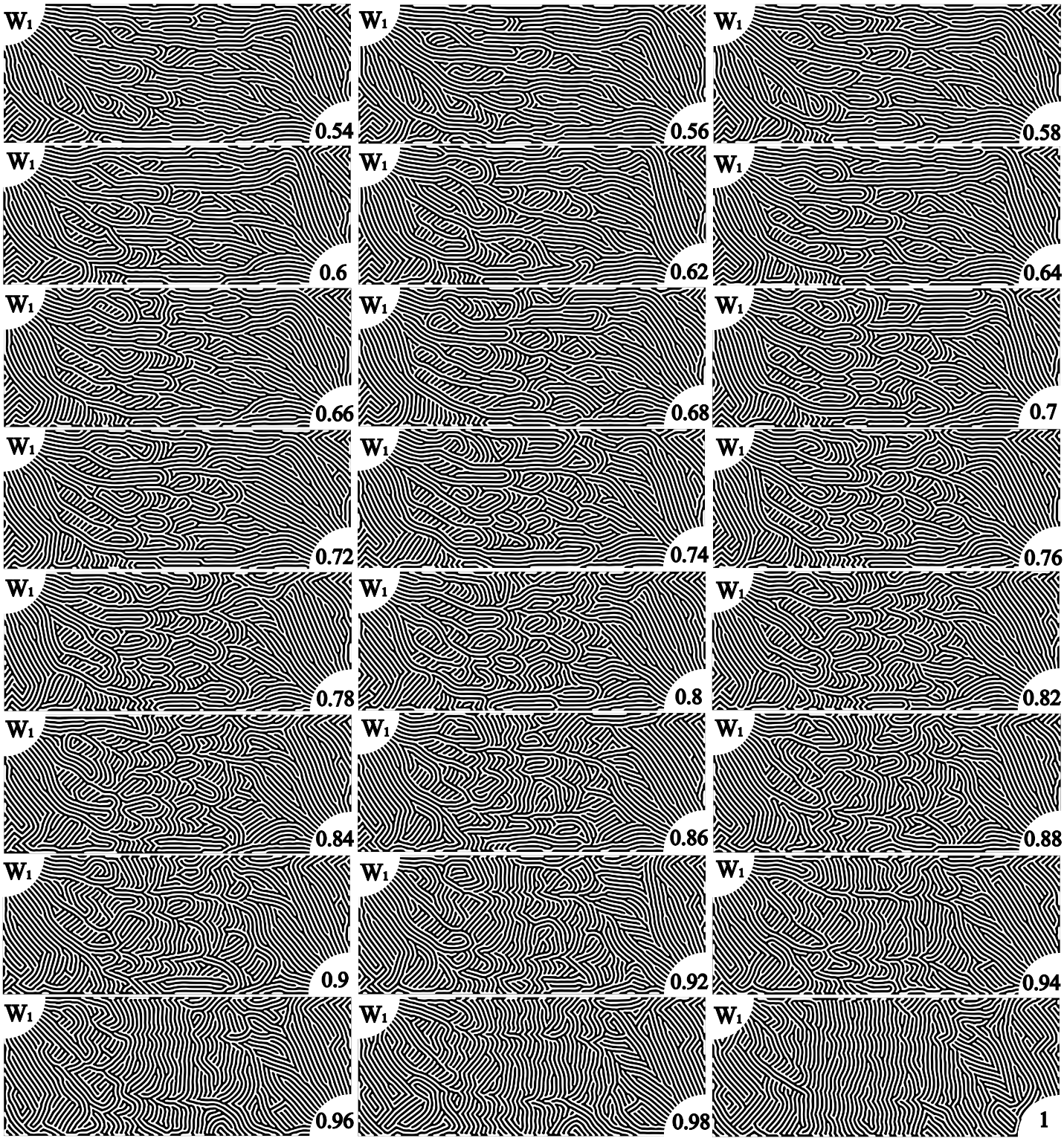
**

**Supplementary D: Diffusion-based dehomogenization of “semi-discrete” design type.**

**
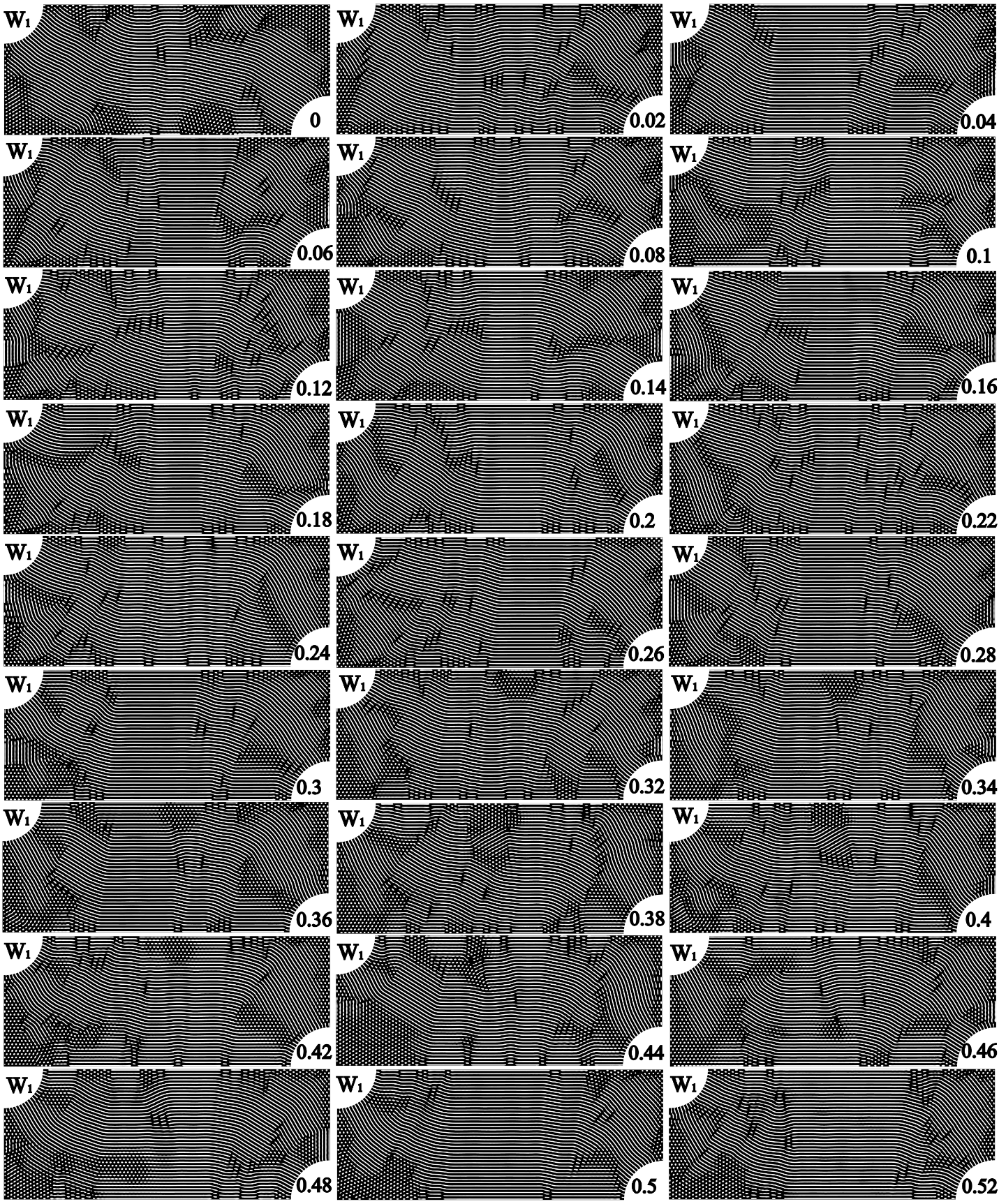
**

**Supplementary D: Continued.**

**
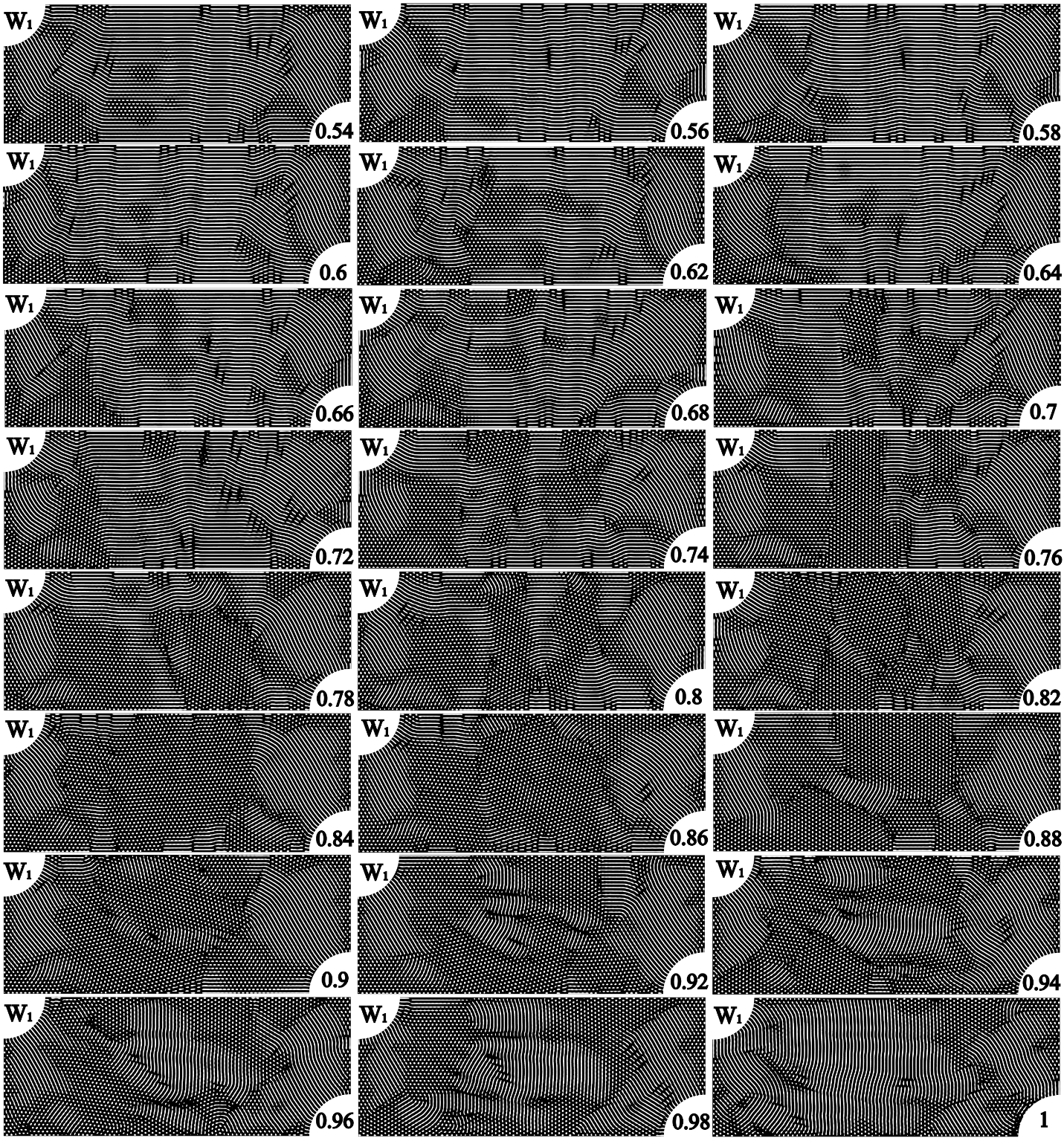
**

**Supplementary E: Homogenization-based optimized orientation fields.**

**
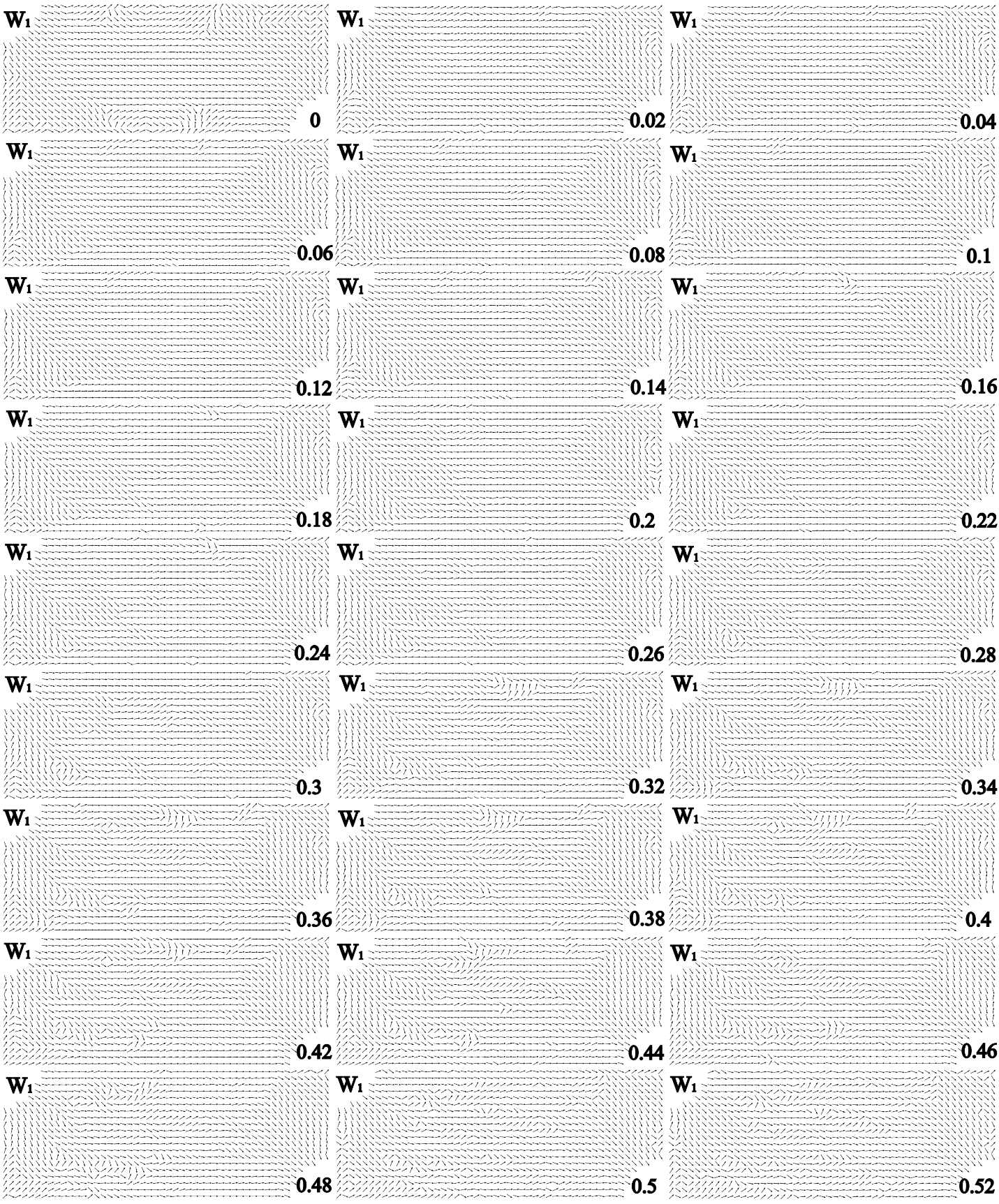
**

**Supplementary E: Continued.**

**
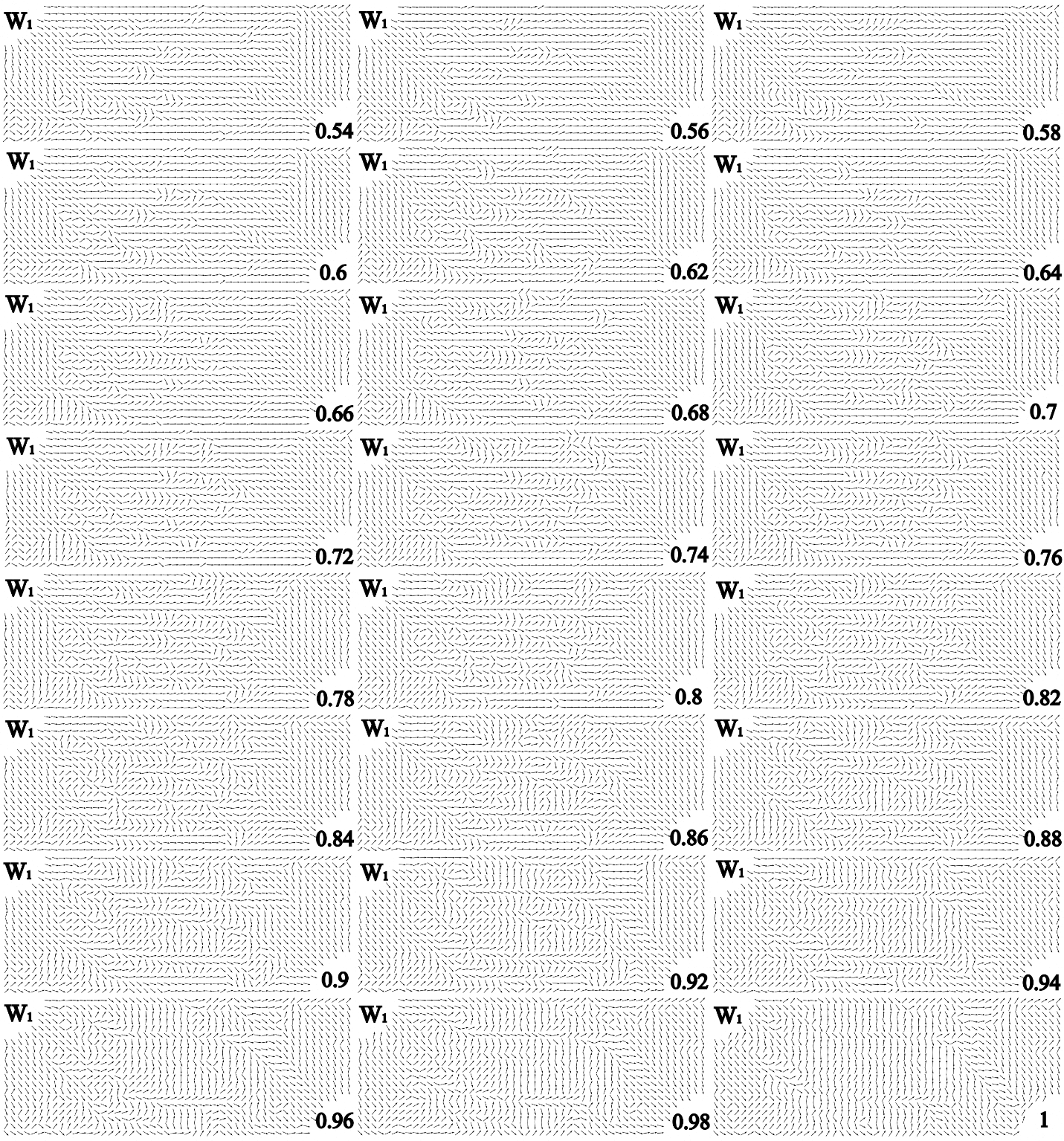
**
